# Editorial: Pathomechanisms and Treatments to Protect the Preterm, Fetal Growth Restricted and Neonatal Encephalopathic Brain

**DOI:** 10.3389/fneur.2021.755617

**Published:** 2021-09-09

**Authors:** Bobbi Fleiss, Deidre M. Murray, S. Tracey Bjorkman, Julie A. Wixey

**Affiliations:** ^1^School of Health and Biomedical Sciences, Royal Melbourne Institute of Technology (RMIT) University, Melbourne, VIC, Australia; ^2^Department of Paediatric and Child Health, University College Cork, Cork, Ireland; ^3^UQ Centre for Clinical Research, Faculty of Medicine, The University of Queensland, Brisbane, QLD, Australia

**Keywords:** preterm (birth), hypoxic-ischemic encephalopathy, fetal growth restriction (FGR), neuroprotection, mechanisms

Every year, many hundreds of thousands of infants are at high risk of perinatal morbidity, poor neonatal outcomes, and life-long disability because they are exposed to events such as preterm birth, fetal growth restriction, or they present with neonatal encephalopathy. Collectively, these negative perinatal events compromise brain development and the high rate of disabilities include outcomes such as cerebral palsy, learning and attention difficulties, behavioral issues, and psychiatric disorders. There are only very limited treatments available to protect the newborn brain. For instance, we are fortunate to have a therapy for neonatal encephalopathy, therapeutic hypothermia, but this is not completely neuroprotective and is difficult to implement outside high resource health care settings. Nevertheless, hypothermia shows that therapies are possible. As such, there is a critical need for further high-quality research into improved identification, stratification, and care of these infants at risk of poor outcomes.

This Research Topic contains an equal contribution of articles focusing on neonatal encephalopathy related to hypoxia-ischemia (HIE) and prematurity, including three systematic reviews examining differing aspects of detection and prediction of adverse neurological outcomes in the HIE infant.

The first systematic review and article of this Topic (Liu et al.) examines the unmet clinical need for markers to predict outcome in hypothermia-treated infants with HIE. The authors consolidate our current knowledge that the currently available clinical tests magnetic resonance imaging (MRI) and electroencephalopathy (EEG) are useful predictors of adverse outcomes, however continued follow-up of the children is crucial to determine whether their predictive abilities include the ability to predict long-term outcomes.

The second systematic review in this issue examined cerebral near infrared spectroscopy (NIRS) monitoring in term and near-term infants with HIE (Mitra et al.). NIRS monitoring is an important tool in the neonatologist's toolbox, as it can provide information regarding changes in cerebral oxygenation and hemodynamics. The authors demonstrated that monitoring HIE newborns using a combination of NIRS and EEG is not only feasible but may improve prognosis of neurodevelopmental outcome. However, further randomized clinical trials and large observational studies are necessary to assess the utility of NIRS in predicting neurodevelopmental outcome and guiding therapeutic intervention.

The importance of defining of a good prognostic marker of adverse neurodevelopmental outcome in HIE babies is also highlighted in the work from Shah et al.. This team showed that in a cohort of HIE babies, increased levels of plasma neurofilament light protein (NfL) were associated with adverse outcomes at 18 months of age in HIE babies who have undergone hypothermia treatment.

A meta-analysis was undertaken to assess the overall strength of the links between birth asphyxia (as determined by pH value of umbilical cord blood) and increased risk of cerebral palsy (CP) by Zhang et al.. This meta-analysis solidified our understanding of the positive association between asphyxia and CP and reassuringly found the available studies are high quality with little publication bias.

The piglet model of HIE has a strong translational value to the human newborn with HIE. In the first of two studies using piglet models of HIE, Pang et al. focus on inflammation sensitized LPS/HIE. They reveal that in the LPS/HIE piglet treated with hypothermia, melatonin, and magnesium, and assessed using magnetic resonance spectroscopy (MRS); that the lactate to N-acetylaspartate (Lac/NAA) ratio is robust outcome predictor—correlating strongly with overall cell death and microglial reactivity.

The second piglet study, (Andelius et al.) induced a relatively mild HIE insult, with a shorter duration of amplitude electroencephalography (aEEG) suppression and duration of hypotension compared to the model from the Robertson team (Pang et al.). Andelius et al. assessed the combined effects of therapeutic hypothermia (TH) and remote ischemic conditioning (RIPC) on HIE. TH itself lowered the lactate to creatine measurement (Lac/Cr) on MRS but there was no additive neuroprotective benefit and additional research in a more severe model is suggested by the authors to fully examine the neuroprotective potential.

We are also provided with specific data on the role of chemokine signaling in driving pathology in LPS-sensitized HIE, using a well-established rat model. Serdar et al., outlined that there is a specific role of NLRP3 upregulation in microglia and the CXCL1/CXCR2 pathway in this model using a battery of gene and protein-based analyses. This pathway may be involved in LPS-sensitized HI brain injury in the newborn and may lead to new treatment options.

A further six articles in this Research Topic examined pathology and mechanisms of brain injury in the preterm born neonate. These are led by an interesting study that supports erythropoietin (EPO) may have neuroprotective abilities for preterm born infants in the context of hyperoxia related injury. Dewan et al. used repeated doses of EPO and showed improvements in short term neuropathology and long-term motor-cognitive outcomes.

Blesa et al. provides evidence for the validity of a novel approach to the analysis of mean diffusivity from 3T MRI, bringing a histogram-based approach validated in adults and showing its promise for assessing preterm born infant brain injury.

Parodi et al. undertook a retrospective cohort study assessing placental and perinatal risk factors for intraventricular hemorrhage (IVH) and cerebellar hemorrhages in preterm infants. Assessing placental pathology may help in understanding mechanisms leading to IVH, though its potential role in predicting cerebellar bleeding needs further investigation.

Although much of the work in this edition focuses on white matter outcomes in preterm born infants, a review from Fleiss et al. outlines the increasingly important evidence for gray matter injury. This review includes a summary of gray matter findings in animal models, how gray matter injury explains neurodevelopmental outcomes and discusses therapeutic targets.

An interesting review presents the hypothesis that in the preterm infant loss of plasma from the circulation results in hypovolemia, and that this is a significant driver of cardiovascular instability and thus poor cerebral oxygenation (Eiby et al.). The authors present their rationale for this hypothesis and how this may be tested in the future.

Further refinements to our imaging arsenal for assessing preterm brain injury were presented by Malova et al.. This retrospective study of 81 patients used susceptibility-weighted imaging (SWI) of punctate white matter lesions in preterm born infants. The study found that are haemosiderin positive SWI lesions occurred more often in infants with lower gestational age and closer to the ventricle. Further research stemming from these interesting findings will hopefully uncover the biological meaning of the observed differences.

Rounding out the edition, is a review from Ross-Munro et al., that proposed that the growth factor midkine may have beneficial roles as a neuroprotectant across forms of perinatal injury. This review brings together a great deal of information on the developmental expression of midkine across species and covers its potential role across a vast number of pathogenic processes.

## Author Contributions

BF and JW wrote the first draft. DM and SB edited the text. All authors (guest associate editors) read and approved the submitted version of the Editorial for this Research Topic.

## Conflict of Interest

The authors declare that the research was conducted in the absence of any commercial or financial relationships that could be construed as a potential conflict of interest.

## Publisher's Note

All claims expressed in this article are solely those of the authors and do not necessarily represent those of their affiliated organizations, or those of the publisher, the editors and the reviewers. Any product that may be evaluated in this article, or claim that may be made by its manufacturer, is not guaranteed or endorsed by the publisher.

